# Emergence of an ancient and pathogenic mammarenavirus

**DOI:** 10.1080/22221751.2023.2192816

**Published:** 2023-04-11

**Authors:** Xue-Lian Luo, Shan Lu, Chuan Qin, Mang Shi, Xiao-Bo Lu, Lu Wang, Sang Ga, Dong Jin, Xin-Li Ma, Jing Yang, Yan Dai, Lin-Lin Bao, Yan-Peng Cheng, Ya-Jun Ge, Yi-Bo Bai, Wen-Tao Zhu, Ji Pu, Hui Sun, Yu-Yuan Huang, Ming-Chao Xu, Wen-Jing Lei, Kui Dong, Cai-Xin Yang, Yi-Fan Jiao, Qi Lv, Feng-Di Li, Jianguo Xu

**Affiliations:** a State Key Laboratory of Infectious Disease Prevention and Control, National Institute for Communicable Disease Control and Prevention, Chinese Center for Disease Control and Prevention, Beijing, People’s Republic of China; b Department of Laboratorial Science and Technology & Vaccine Research Center, School of Public Health, Peking University, Beijing, People’s Republic of China; c Research Units of Discovery of Unknown Bacteria and Function, Chinese Academy of Medical Sciences, Beijing, People’s Republic of China; d Department of Epidemiology, School of Public Health, Shanxi Medical University, Taiyuan, People’s Republic of China; e Key Laboratory of Human Disease Comparative Medicine, Chinese Ministry of Health, Beijing Key Laboratory for Animal Models of Emerging and Remerging Infectious Diseases, Institute of Laboratory Animal Science, Chinese Academy of Medical Sciences and Comparative Medicine Center, Peking Union Medical College, Beijing, People’s Republic of China; f The Center for Infection & Immunity Study, School of Medicine, Shenzhen campus of Sun Yat-sen University, Shenzhen, People’s Republic of China; g Infectious diseases department, First Affiliated Hospital, Xinjiang Medical University, Urumqi, People’s Republic of China; h Kashi Center for Disease Control and Prevention, Kashi, People’s Republic of China; i Yushu Prefecture Center for Disease Control and Prevention, Yushu, People’s Republic of China; j Kashi first people’s hospital, Kashi, People’s Republic of China; k Institute of Public Health, Nankai University, Tianjin, People’s Republic of China

**Keywords:** Mammarenavirus, evolution history, ancient evolution, pathogenicity, plateau pika

## Abstract

Emerging zoonoses of wildlife origin caused by previously unknown agents are one of the most important challenges for human health. The Qinghai-Tibet Plateau represents a unique ecological niche with diverse wildlife that harbours several human pathogens and numerous previously uncharacterized pathogens. In this study, we identified and characterized a novel arenavirus (namely, plateau pika virus, PPV) from plateau pikas (*Ochotona curzoniae*) on the Qinghai-Tibet Plateau by virome analysis. Isolated PPV strains could replicate in several mammalian cells. We further investigated PPV pathogenesis using animal models. PPV administered via an intraventricular route caused trembling and sudden death in IFNαβR^-/-^ mice, and pathological inflammatory lesions in brain tissue were observed. According to a retrospective serological survey in the geographical region where PPV was isolated, PPV-specific IgG antibodies were detected in 8 (2.4%) of 335 outpatients with available sera. Phylogenetic analyses revealed that this virus was clearly separated from previously reported New and Old World mammarenaviruses. Under the co-speciation framework, the estimated divergence time of PPV was 77–88 million years ago (MYA), earlier than that of OW and NW mammarenaviruses (26-34 MYA).

## Introduction

The emergence of novel infectious diseases such as severe acute respiratory syndrome coronavirus 2 (SARS-CoV-2), pandemic influenza and monkeypox are serious threats to public health and global security [1–3]. More than 60% of human emerging infectious diseases are zoonotic, indicating that viruses that originate in wild mammals are of particular concern [4,5]. Therefore, understanding of viral diversity in wild mammals, viral host range and cross-species transmission or spillover are key goals in pandemic surveillance [4].

Mammarenaviruses are predominantly zoonotic pathogens with rodent vectors, several of which are highly virulent and handled in Biosafety Level 4 laboratories, including Guanarito virus, Junin virus, Machupo virus, Lassa virus (LASV) and Sabia virus [6]. Based on phylogenetic, serological, and geographical differences, mammarenaviruses are divided into two groups: Old World (OW) and New World (NW) [7,8]. The OW group is associated with Eurasian rodents in the family *Muridae*, and the NW group is associated with American rodents in the subfamily *Sigmodontinae*. Lymphocytic choriomeningitis virus (LCMV) belongs to the OW group and has the widest known distribution of any other mammarenavirus because of its widespread host (*Mus musculus*) [9]. In China, Wenzhou virus (WENV), a newly discovered mammarenavirus in rodents, has been reported to be associated with disease in humans [10,11]. It was hypothesized that the NW and OW mammarenaviruses share a common ancestor that chronically infected a common rodent ancestor before New World sigmodontine and Old World murids diverged, approximately 35 million years ago [6].

The Qinghai-Tibet Plateau is the highest plateau in the world, 4,500 m above sea level on average, and represents a unique ecological niche with diverse wildlife [12–14]. Here, we discovered and characterized a novel mammarenavirus designated plateau pika virus (PPV) from the most prevalent lagomorph (plateau pika) in the Qinghai-Tibetan Plateau. The evolutionary history and pathogenicity of PPV were further explored.

## Materials and methods

### Ethics committee

The trapping and investigation of plateau pikas were approved by the Ethics Committee of the National Institute of Infectious Diseases Control and Prevention of China CDC (No. ICDC-2016004). The collection of retrospective human sera was approved by the Committee on the Ethics of National Institute of Infectious Diseases Control and Prevention of China CDC (No. ICDC-2019012).

### Wild animal virome study

**Figure 1.**
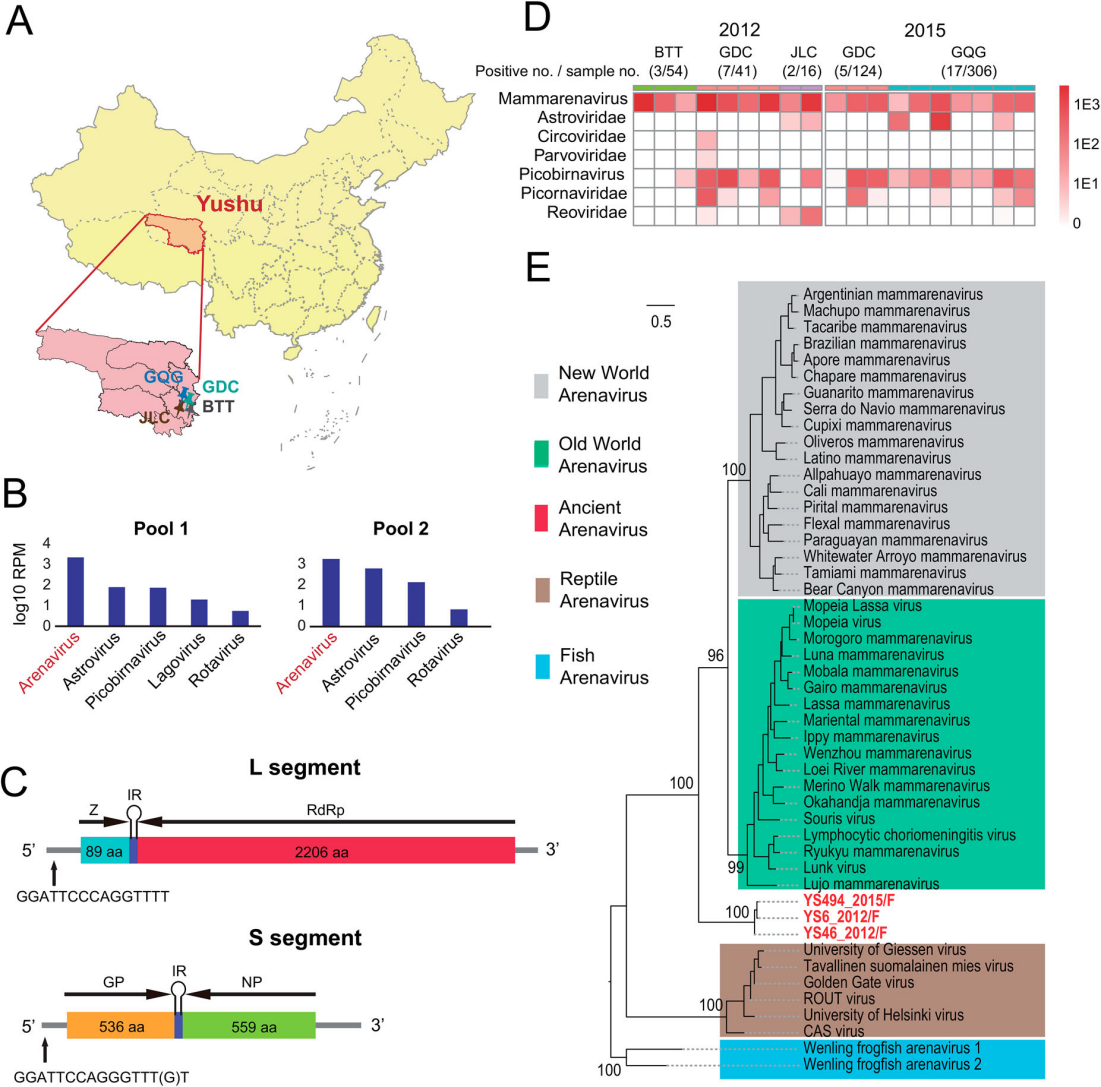
**A novel arenavirus discovered in plateau pikas.** (A) A map showing the location of sampling sites in Yushu, Qinghai Province, China. Samples were collected from the GQG, GDC, BTT and JLC counties. (B) The dominant mammalian virus discovered in the intestinal contents of plateau pikas by RNA-seq. (C) A schematic of the genome organization of PPV. (D) The distribution of PPV and the abundance of the dominant mammalian virus from plateau pikas in 2012 and 2015. Darker colour reflects higher virus abundance. (E) Phylogenetic analysis of PPV and other arenaviruses. PPVs are marked with red.

*Ochotona curzoniae* (commonly known as “plateau pika”) was captured in Yushu, Qinghai Province, China, in four locations: Guoqinggou (GQG; with an altitude of 3599 m above sea level, 96.4°E, 33.1°N), Gandacun (GDC; 3935 m above sea level, 96.8°E, 33.1°N), Batangtan (BTT; 3931 m above sea level, 97.1°E, 32.8°N), and Jielachong (JLC; 3970 m above sea level, 96.5°E, 33.4°N) (Figure 1(A)). Captured individuals were immediately transported to the laboratory, euthanized by intracardiac delivery of sodium pentobarbitone and dissected. After euthanasia of the animals, internal organs (e.g. kidneys, lungs, heat, spleen, brain and liver) as well as gut content were harvested for each individual. These samples were flash frozen and kept in liquid nitrogen before they were subsequently transferred and stored in −80 °C freezers.

### Virus discovery

RNA was extracted from fecal samples, which were subsequently processed in pools (size: 50 samples) or individually using library construction and sequencing procedures as previously described [13]. Briefly, the RNA was subjected to rRNA removal using the Next Poly (A) mRNA Magnetic Isolation Module (NEB, USA) and total RNA library construction using the Next Ultra® RNA Library Prep Kit (NEB, USA). The subsequent 150 bp paired-end libraries were sequenced using the Illumina HiSeq 4000 platform. The resulting reads were assembled into contigs using the Trinity programme [15]. Virus identification was carried out by blasting contigs against the nonredundant protein database (nr) and searching for virus hits. Taxonomic annotation was then performed on each of the top hits. The genome segments of the family *Arenaviridae* were confirmed by mapping reads to the corresponding contigs as well as by RT–PCR and Sanger sequencing (Table S1). The termini of these genome segments were determined using the 5'/3’ RACE kits (Roche, Germany).

### Evolutionary analyses

For newly identified plateau pika arenavirus, ZP, RdRp, GP, and NP viral proteins were predicted from the corresponding genome segments. These proteins were aligned and compared with those derived from all existing members (species) of the family *Arenaviridae* using the L-INS-i algorithm implemented in the MAFFT programme [16]. Poorly aligned regions and major gaps were subsequently removed from the alignment using the trimal programme [17]. Based on these alignments, phylogenetic trees were reconstructed with the maximum likelihood algorithm implemented in the phyML programme v3.0 [18], for which an LG+Γ amino acid substitution model and a subtree pruning and regrafting (SPR) branch-swapping algorithm were used. The support for the internal nodes in the phylogenetic trees was derived from an approximate likelihood ratio test (aLRT) with the Shimodaira-Hasegawa-like procedure. Finally, the extent of virus–host codivergence was examined by an event-based co-phylogenetic reconstruction approach implemented in the Jane programme (version 4) [19], where the virus tree was derived from RdRp phylogeny and the corresponding host tree was obtained from the TIMETREE website (http://www.timetree.org/). The cost scheme was set as follows: codivergence = 0, duplication = 1, host switch = 2, loss = 1, failure to diverge = 1. The number of generations and the population size were both set to 100. The significance of codivergence was evaluated by comparing the estimated costs to null distributions derived from 100 randomizations of tip mapping.

### Virus isolation and growth curve

Rabbit kidney (RK-13) cells were cultured at 37 °C in MEM with 10% FBS, 100 U/mL penicillin, and 100 μg/mL streptomycin. One hundred milligrams of lung or liver tissue from virus RNA-positive plateau pikas was lysed in 1 mL of PBS by Tissue Lyser II (Qiagen, Germany), and inoculated onto the cells for 1 h. The supernatant was then removed. The cells were washed, and incubated in MEM with 2% FBS. CPE was observed every day. The cells were blindly passaged three times. The study was conducted in the biosafety level 3 lab of the National Institute for Communicable Disease Control and Prevention, Chinese Centre for Disease Control and Prevention.

For analysis of the kinetics of PPV replication in RK-13, African green monkey kidney cells (Vero), human lung cancer cells (A549), and human embryo lung fibroblast diploid cells (SLF-1, patent no. CN103255102), confluent cell monolayers in 24-well plates were treated with PPV (MOI  = 0.5) per well with three replicates for each cell line. Inoculation was performed as described above. After inoculation, the cells were frozen three times and then centrifuged at 1000×g for 3 min. The supernatant was harvested at 10, 24, 48 and 72 h and PPV RNA levels were tested by qRT–PCR.

### Electron microscopy

The 100-mL virus-infected cell supernatant was harvested at passage four (P4). The supernatant was centrifuged at 6,000×g for 30 min to remove cell debris. The supernatant was then ultracentrifuged at 10,0000×g for 1 h. The precipitate was dissolved overnight in PBS with 2% formaldehyde and added to copper. After negative staining with phosphotungstic acid, the virus particles were observed under a transmission electron microscope (F20; FEI). The cell pellets were also subjected to the transmission electron microscope (F20; FEI) as described [10].

### Animal experimental infection

To find suitable laboratory animal models for PPV infection, we tested 3-month-old New Zealand white rabbits, 6-week-old interferon alpha/beta receptor-deficient mice (IFNαβR^-/-^ mice), 7-day-old suckling mice and 6-week-old SPF BALB/c mice. All animals were inoculated with 50 µL of virus (10^5^TCID_50_/mL) in PBS. Administration routes varied by model: 1) multipoint subcutaneous and intravenous infection was used for New Zealand white rabbits; 2) intraventricular and intraperitoneal infection for IFNαβR^-/-^ mice and suckling mice; and 3) intraventricular and intravenous infection for SPF BALB/c. Mock groups (N = 3) were inoculated with sterile PBS through similar administration routes. Signs of disease, weight loss, and mortality were monitored every day up to 14 days post-infection (d.p.i).

For further characterization of the pathogenesis of PPV in IFNαβR^-/-^ mice, three groups (N = 6 per group) of 6-week-old IFNαβR^-/-^ female mice were intraventricularly inoculated with 50 µL of virus (10^5^ TCID_50_/mL) in PBS. Mock groups (N = 3) were inoculated with sterile PBS. The cerebrum, cerebellum, spinal cord, and other organs (kidney, lung, heart, spleen, and liver) were harvested and examined at 0, 3, 5 and 7 d.p.i. The tissues were fixed with 10% paraformaldehyde, sectioned, and stained with hematoxylin and eosin (H&E). Samples of the brain, kidney, lung, heart, spleen, and liver were also detected for viral nucleic acids at 3, 5, and 7 d.p.i. This study was conducted in an animal biosafety level 3 containment unit of the Institute of Laboratory Animal Science, Chinese Academy of Medical Sciences [20].

### Specific qRT–PCR of PPV RNA

Viral RNA extracted from all types of samples from plateau pikas, cell culture supernatants, IFNαβR^-/-^ mice and human sera was quantified by quantitative reverse transcription-PCR (qRT–PCR) based on the partial L gene of PPV using the one step real-time RT–PCR (TaKaRa, Japan) with the following primers (F-L1: 5'TACCTCTGCAGGAAGTGCCA3’; R-L1: 5'CCCACTGGGTGGGTTAGTCA3’; 4Probe-L1: 5'-FAM-ACAGCCCCACCTGTTGTGTCTGTGGGA-BHQ1-3’). The reaction was performed at 50 °C for 30 min and 95 °C for 3 min, followed by 40 cycles of 95 °C for 30 s and 55 °C for 30 s.

Viral copies were calculated using standard curves of serially diluted standard target RNA, which was an in vitro transcription product of a plasmid containing the 126-bp RdRp region.

### Indirect immunofluorescence analysis (IFA)

Serological screening of human serum samples was initially performed by IFA using PPV-infected cells. A total of 335 human sera from outpatients were available from the surveillance programme for influenza virus infection in Yushu, Qinghai Province. Briefly, RK-13 cells were inoculated with PPV. After 3 days of inoculation, cells were harvested and fixed on glass slides with 4% paraformaldehyde in PBS for 1 h at 4 °C. Serum samples were diluted to 1:20 prior to inoculation with infected RK-13 cells. Goat anti-human fluorescein isothiocyanate (FITC)-labeled antibody (1:200) was used as a secondary antibody. Images were obtained by a fluorescence microscope (Revolve Gen 2; Echo).

### Western blot analysis

Recombinant NPs from PPV, WENV and LCMV were expressed in the Bac-to-Bac Baculovirus Expression System (Invitrogen, CA, USA) as previously described with some modifications [21]. Briefly, NP genes from PPV [933 bp (748 nt-1680 nt), GenBank accession no. MN443996], WENV (1704bp, GenBank accession no. KM051422), and LCMV (1680 bp, GenBank accession no. DQ286931) were amplified by pGEX-4T-NP and synthesized by Beijing Rui Biotech Co., Ltd. (Beijing, China). cDNA of NPs was then cloned into the plasmid pFast HTa vector (Invitrogen) to generate pFast/NPs. Recombinant bacmids were produced following transformation of the recombinant plasmid pFast/NPs into *Escherichia coli* DH10Bac competent cells (Invitrogen) according to the manufacturer’s protocol. The recombinant bacmid DNAs were extracted from *E. coli* DH10Bac and transfected into Sf9 cells using CellFectin reagent (Invitrogen) to yield recombinant baculovirus AcMNPV/NPs. For purified NPs, Sf9 cells were infected with AcMNPV/NPs at a multiplicity of infection (MOI) of 5. Three days post-infection, the cells were harvested, resuspended in lysis buffer (20 mM Tris–HCl, 0.2 M NaCl, 1 mM EDTA, pH 7.4, 10 mM β-mercaptoethanol) and then sonicated for 10 min at 300 W. Cell debris was removed by centrifugation for 30 min at 11,000 × g. The NP proteins in the supernatant were purified using His·Bind® Purification Kit Ni-NTA affinity chromatography [22] and confirmed by western blot analysis using anti-6×His antibody (Suzhou Biodragon Immunotechnologies Co.,Ltd, Suzhou, China).

Purified NPs were separated by 12% SDS–PAGE gels and transferred to a nitrocellulose membrane (Pierce, Rockford, IL). The membranes were blocked for 1 h at room temperature with 5% nonfat milk in Tris-buffered saline (150 mM NaCl, 100 mM Tris–HCl, pH 7.6) containing 0.1% Tween-20 (TBST). Following blocking, membranes were incubated overnight at 4 °C with human sera (1:200) in blocking buffer. Goat anti-human HRP-labeled antibody (1:10,000) was used as a secondary antibody (Suzhou Biodragon Immunotechnologies Co.,Ltd, Suzhou, China). Membranes were scanned by an Amersham Imager 680 (GE, CT, USA).

Mice were inoculated with the recombinant antigens from *E. coli* to produce antisera against PPV, WENV and LCMV NP. Antigen cross-reactivity against NP from PPV, WENV and LCMV was assessed by western blot assays.

## Supplementary Material

Supplemental Material

## Figures and Tables

**Figure 2.**
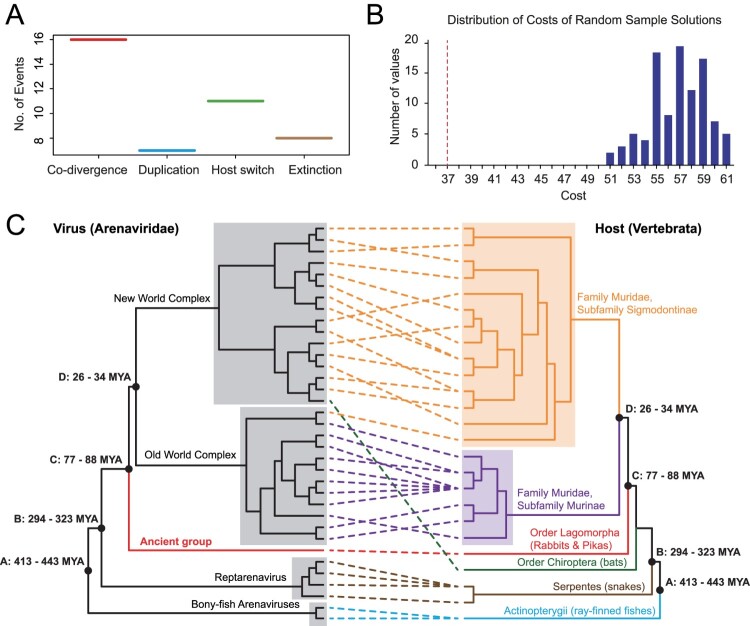
**Co-divergence analyses of arenaviruses and their associated hosts.** (A) Distribution of estimated co-divergence and non-codivergence (e.g. duplication, host switching, and extinction) events. (B) Estimated sum of non-codivergence events (red dotted line) in comparison to the associated null distributions under 100 randomizations of virus–host associations. (C) Comparisons of the topologies of virus and host phylogenetic trees. Well-established co-divergence points (black solid dots) are marked on the node. For these nodes, the divergence times of the host groups were used to calibrate the divergence times of the corresponding virus groups.

**Figure 3.**
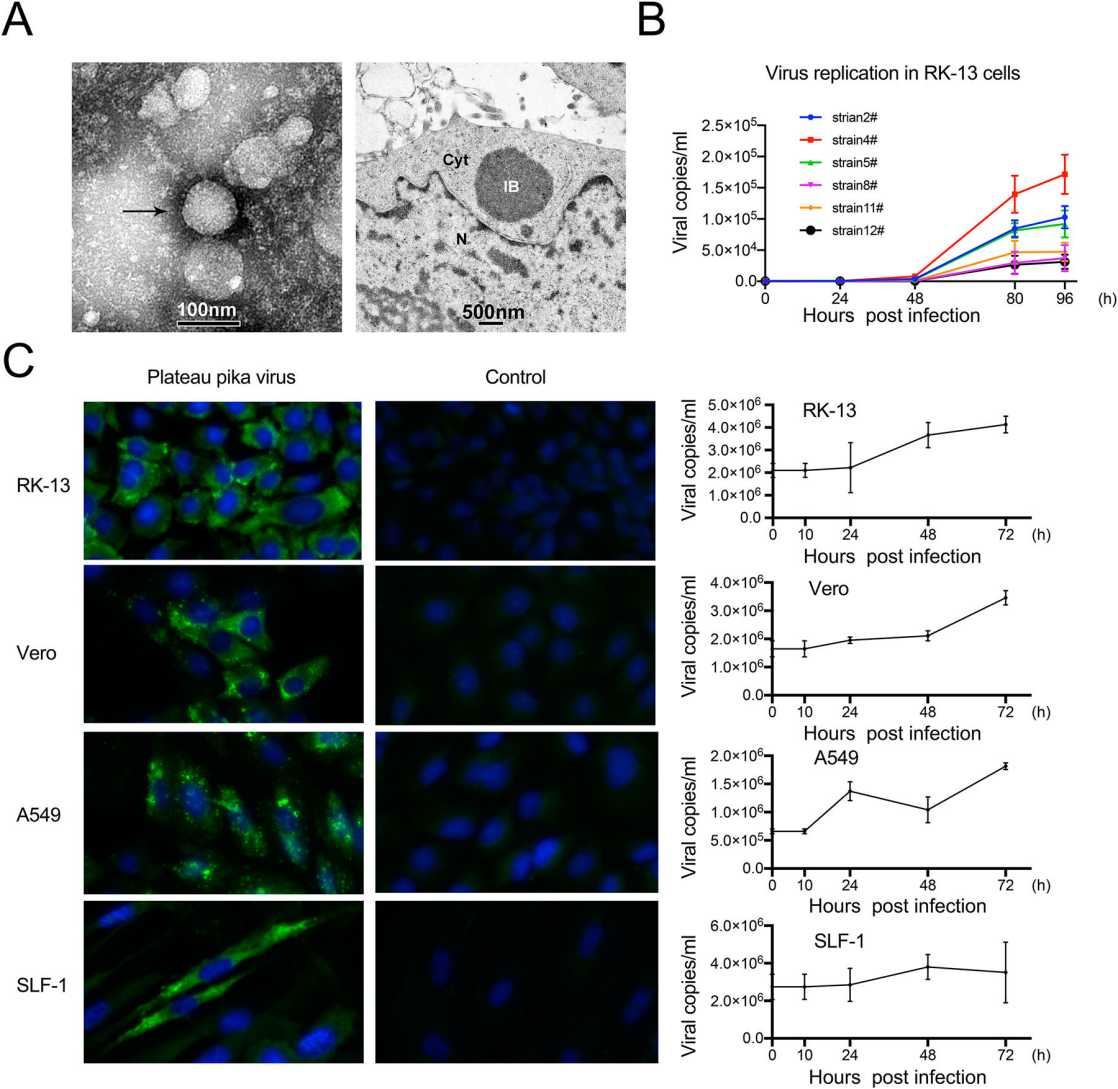
**Biological features of PPV.** (A) Electron microscope images of PPV in cell culture supernatant (arrow, left) and in infected RK-13 cell (IB, right). IB: inclusion body. N: nucleus. Cyt: cytoplasm. (B) The growth of six isolates of PPV in RK-13 cells. RK-13 cells infected with PPV (MOI  = 0.5) for 24, 48, 80 and 96 h. PPV RNA levels were measured by qRT–PCR. Values are the averages of three independent experiments (mean ± SD). (C) The replication of PPV in different cell lines. RK-13, Vero, A549 and SLF-1 cells were infected with PPV (MOI  = 0.5) for 10, 24, 48 and 72 h, and PPV RNA levels were measured by qRT–PCR. Values are the averages of three independent experiments (mean ± SD).

**Figure 4.**
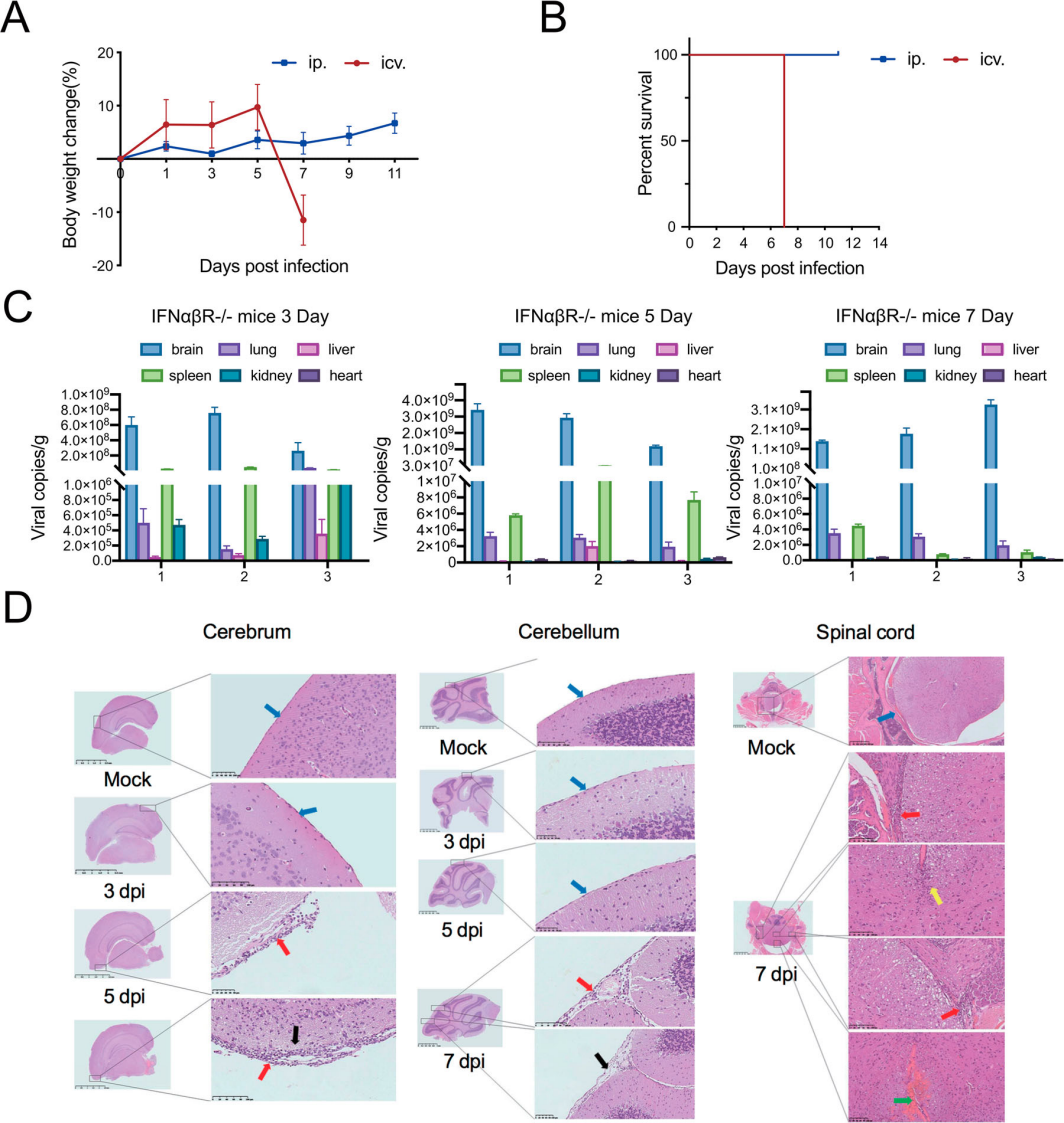
**Histopathological analyses of IFNαβR^-/-^ mice intraventricularly infected with PPV.** (A) Weight loss of IFNαβR-/-mice infected with PPV via intraperitoneal (ip) and intraventricular (icv) infection. (B) The death rate of IFNαβR-/-mice infected with PPV via intraperitoneal (ip) and intraventricular (icv) infection. (C) Viral load in different tissues of IFNαβR-/-mice infected with PPV at 3, 5, and 7 days post-infection (d.p.i.). (D) Histopathological changes in the cerebrum, cerebellum and spinal cord of mice intraventricularly challenged with PPV. Blue arrows indicate the enlargement of normal areas. Red arrows indicate the inflammatory infiltrates. The black arrow indicates cortical edema. Yellow arrows indicate gliosis in the spinal cord. Green arrows indicate hematomyelia. [Cerebrum scale bars: 0.5 mm (left); 20 μm (right); cerebellum scale bars: 0.2 mm (left); 20 μm (right); spinal cord scale bars: 0.2 mm (left); 20 μm (right).]

**Figure 5.**
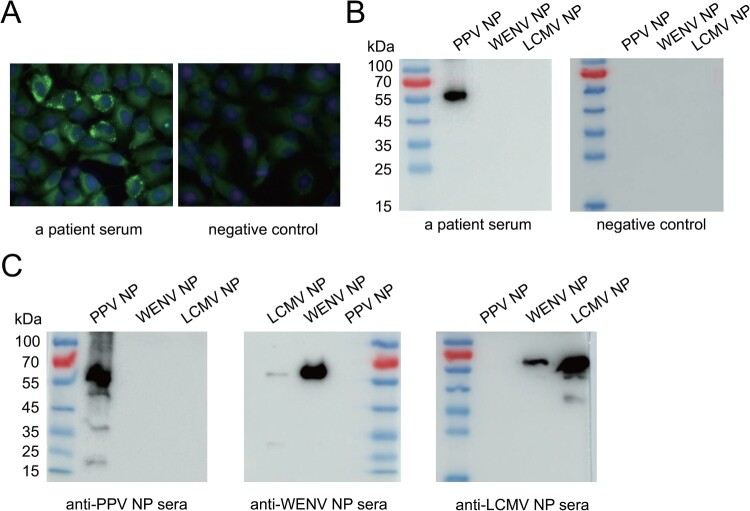
**Evidence of human exposure by IFA and western blot.** (A) Representative immunofluorescence images of a serum sample from a patient (left) and from a nonexposed individual (right). Human serum samples were diluted 1:40. (B) Representative western blot images of a serum sample from a patient (left) from a healthy individual (right). Purified NPs (400 ng) were used as antigens. Nonexposed human serum was used as a negative control. Human serum samples were diluted 1:200. (C) Cross-reactivity between PPV and WENV/LCMV NPs. Mouse antisera against PPV NP, WENV NP and LCMV NP were diluted at 1:2000 and incubated with the PPV, WENV and LCMV NPs, respectively. The loading amount of NP for each lane was 400 ng.

**Table 1.** Details of sera screening for PPV.

Collection locations	Collection dates	Patient type	Number of samples	Clinical signs	Mean age in years (range)	95% CI (years)	Test used	Number (proportion) of positive
Yushu, Qinghai	2012	Outpatients	235	Influenza-like illness	NA	NA	IFA、WB	5 (2.1%)
Yushu, Qinghai^a^	2019	Outpatients	100	Influenza-like illness	11.0(7 years to 34 years)	9.0-14.0	IFA、WB	3 (3%)

NA: not available

^a^ Batang county where PPV was isolated in Yushu, Qinghai.

## Data Availability

The genome sequences of PPV are available from GenBank (accession numbers MN443982-MN444000 and MN444002-MN444020).

## Results

### Identification of a novel arenavirus in plateau pikas

A total of 113 fecal samples were collected and grouped into two pools for meta-transcriptomic sequencing. Data analyses revealed that arenavirus-associated contigs had the highest abundance levels, although other viruses such as astroviruses, picobirnaviruses, and rotaviruses were also discovered in both pools (Figure 1(A) and (B)). For each pool, both ambisense L and S segments were identified, which encoded zinc-binding RING protein (ZP) (89 aa), RNA-dependent RNA polymerase (RdRp) (2,206 aa), glycoprotein (GP) (536 aa), and nucleoprotein (NP) (559 aa). The final lengths for the L and S segments were determined to be 7106 and 3540 bases, respectively, by 5’ and 3’ RACE. There were 15 bp conserved reverse complementary sequences, namely, GGATTCCCAGGTTTT and GGATTCCAGGGTTT(G)T, at both the 5’ and 3’ termini of the L and S segments, respectively (Figure 1(C)). We designated this newly identified arenavirus plateau pika arenavirus (PPV).

### Distribution and tissue tropism of PPV arenaviruses

We surveyed the distribution of new PPV arenaviruses in the plateau pikas from BTT, GDC and JLC in 2012 and from GDC and GQG in 2015. The data showed that PPV RNA was detected in 34 (6.3%) of 541 intestinal content samples from these counties (Figure 1(D)). Nineteen positive samples (9 samples collected in 2012, and 10 samples collected in 2015) were randomly selected for RNA-seq sequencing (Table S2). Evolutionary divergence analysis between these sequences by MEGA7.0 showed 27∼39 (RdRp), 30∼33 (NP), 31∼37 (GP) and 26∼39 (ZP) amino acid identity (%) to known mammarenavirus (Table S3). This relationship among the PPV sequences was supported by the highest identities between each other, with 82∼100 nucleotide identity (%) to the L and S segments (Table S4). The data showed that PPV was the dominant virus among the identified mammalian viruses (Figure 1(D)). PPV RNA was detected in infected plateau pikas in the lungs (100%), kidney (97%), heart (96%), liver (94%), and spleen (96%) (Table S5); brain samples were not collected. Viral loads in the tested organs of plateau pikas ranged from 7 × 10^2^ to 6 × 10^7^ copies/g, and the lung showed the highest concentration of viral RNA (Figure S1). These results indicated that plateau pikas were a natural host of PPV.

### Evolutionary history of PPV arenaviruses

Previous reports showed that the family *Arenaviridae* contained 4 clades, including two mammarenaviruses (Old World and New World), one associated with reptiles and one with fish [23]. We further investigated the evolutionary history of PPV arenaviruses within the context of the entire *Arenaviridae* family (Figure 1(E)). Interestingly, the newly identified PPV arenavirus formed a divergent sister clade to both Old World and New World mammarenaviruses. Nevertheless, it shared much closer relationship with members of mammarenavirueses (33∼37% amino acid identity at RdRp) than reptile (27%) or fish arenaviruses (30%). This phylogenetic position, as well as the fact that the virus was identified from plateau pika, a mammalian species, suggests that it is more likely to be a divergent member of mammarenavirus. Furthermore, this topology was also supported by phylogenies constructed based on nucleoprotein and glycoprotein (Figure S2), although for glycoproteins the fish and reptile counterparts are too divergent for sequence alignment and therefore are not included in the tree (Figure S2).

To estimate the divergence time of PPV arenaviruses from the other arenaviruses, we first reconciled the phylogenetic history of viruses with that of their hosts, with the assumption that if the virus co-speciate with the host, the host divergence time could be used on the virus tree. Our reconciliation analyses with the programme Jane 4 revealed 16 co-speciation events and 26 noncospeciation event (duplications = 7; extinction = 8; host-jumping = 11, Figure 2(A)). The total cost estimated was 37, which was significantly less than that expected by chance (*p* < 0.01) and therefore strongly favoured the co-speciation hypothesis (Figure 2(B)). Under the co-speciation framework, the estimated divergence time of the ancient group was 77–88 million years ago (MYA), earlier than that of Old World and New World mammarenaviruses (26-34 MYA) (Figure 2(A-C)). The ancient group was proposed (Figure 1(E), Figure 2(C)).

### Isolation and growth properties of PPV arenaviruses

We successfully isolated PPV strain #4 from the lung tissue of plateau pikas. PPV strain #4 was stably passaged in RK-13 cells but caused no visible CPE. Negative-stain electron microscopy showed an 80-nm round particle with an outer layer formed by surface projections, indicating that PPV had morphologic similarities to viruses in the genus mammarenavirus (Figure 3(A)). Inclusion bodies of approximately 1 μm in diameter were observed in the infected RK-13 cells by transmission electron microscopy (Figure 3(A)). Subsequently, we isolated five additional strains from lung and liver tissues of plateau pikas. After sequence alignment, these isolates shared 85∼98% (for the L segment) and 82%∼99% (for the S segment) nucleotide identity with each other (Table S6). All isolates replicated in RK-13 cells, of which strain #4 seemed slightly more efficient than the other isolates (Figure 3(B)). Hence, we focused on strain #4 for further study including the growth properties and pathogenicity of PPV.

We further investigated cell susceptibility to PPV using cell lines originating from nonhuman primates (Vero) and humans (A549, and SLF-1). No visible CPEs were observed in these three cell lines, which was consistent with observations in RK-13 cells. IFA showed that PPV could replicate in Vero, A549, and SLF-1 cells (Figure 3(C)). Furthermore, qRT–PCR showed that PPV replication in Vero and SLF-1 cells was more efficient than that in A549 cells, with viral loads increasing by 2.0 × 10^6^ copies/mL in the supernatant (Figure 3(C)).

### The histopathologic lesions in brain tissues of mice

We then conducted animal experiments to investigate the potential pathogenesis of PPV using multiple mouse genotypes, and New Zealand white rabbits. The body weights of SPF BALB/c mice and New Zealand white rabbits decreased transiently but recovered quickly (Figure S3). We also found that the body weights of suckling mice and IFNαβR^-/-^ mice continually increased throughout the observation period after intraperitoneal infection (Figure S3). However, the body weights of IFNαβR^-/-^ mice infected intraventricularly did not change until 5 d.p.i. but showed a steep drop at 7 d.p.i. (Figure 4(A)). At this time, clinical observation included limb weakness, trembling of the whole body and sudden death (Figure 4(B)).

Therefore, IFNαβR^-/-^ mice were chosen as the model for further analysis. The mice were infected by intraventricular PPV injection. The animals were then euthanized. The brain, spinal cord, heart, liver, spleen, lung and kidney of intraventricularly inoculated mice were collected for viral load and pathological analysis at 0, 3, 5, and 7 d.p.i. (n = 3) respectively (Figure 4(C) and (D)). All organs tested were positive for PPV, and the brain had the highest viral load. The viral loads in the brain, lung, spleen and heart increased at the initial 5 d.p.i and maintained a high level at 7 d.p.i (Figure 4(C)). H&E staining of the tissue sections showed that infection with PPV led to inflammatory infiltrates in the cerebral cortex at 5 d.p.i, and further progressed to cortical edema by 7 d.p.i. (Figure 4(D)). The cerebellar cortex showed inflammatory infiltrates and cortical edema on 7 d.p.i. (Figure 4(D)). IFNαβR^-/-^ mice infected with PPV displayed a small amount of inflammatory cell infiltration around the spinal meningeal vessels and the nerve root of the spinal cord, with proliferation of glial cells and hemorrhage in the spinal cord at 7 d.p.i (Figure 4(D)). No obvious lesions were observed in the heart, liver, spleen, lung, or kidney at 7 d.p.i (Figure S4).

### Serological evidence of PPV infection in humans

We further investigated potential PPV infection cases in Yushu, Qinghai Province where PPV was isolated. Serological screening of human sera was initially performed by IFA using PPV-infected cells. A total of 335 sera from outpatients in Yushu were screened for anti-PPV IgG antibodies, with 8 (2.4%) samples testing positive (Figure 5(A), Figure S5(A)). In addition, we conducted specific detection of anti-PPV IgG antibodies by western blot assay using purified nucleoprotein of PPV. Consistent with the IFA results, WB showed that the same 8 (2.4%) samples, out of the 335 tested were positive (Figure 5(B), Figure S5(B)). The PPV IgG WB assay was used with LCMV- and Wenzhou virus-positive mouse sera (Figure 5(C)). There was obvious cross-reactivity between LCMV and WENV, but no serological cross-reactivity was observed between PPV and LCMV/WENV (Figure 5(C)). Thus, PPV-specific IgG antibodies were detected in 8 (2.4%) of 335 outpatients with available sera (Table 1).

## Discussion

Our virome study of plateau pikas in the Qinghai-Tibet Plateau identified a new mammarenavirus, named plateau pika virus (PPV). PPV has the same genome structure and protein-encoding form as existing members of mammarenavirus. Nevertheless, it shared less than 40% amino acid identity at RdRp with other known mammarenaviruses (Table S3). A phylogenetic analysis based on the amino acid sequences of the RdRp protein encoded by PPV using reptarenaviruses and hartmaniviruses as outgroups suggested that PPV is distinct from the known NW and OW mammarenaviruses and is classified as an ancient group of the genus Mammarenavirus (Figure 1(E)). Following LCMV and WENV, PPV was the third arenavirus reported and isolated in Asia. Nevertheless, despite this discovery, the diversity of non-rodent mammarenaviruses is still limited, and substantial gaps remain between mammarenaviruses and reptarenaviruses, which need to be filled by further study of arenaviruses from diverse mammalian host groups.

Our study provides key evidence that demonstrates the ancient history of the family *Arenaviridae*, which is mainly reflected by the high resemblance of virus and host phylogenies (Figure 2). Such co-divergence theory was initially proposed for arenaviruses because of the observation that each arenavirus species is associated with a single rodent host species and that the virus and host share matching phylogeographic history [6,24]. However, a later co-phylogenetic analysis based on 49 mammarenavirus and their associated host suggested that the hypothesis of virus–host co-speciation is unlikely to hold. The divergence of Old World and New World arenaviruses is estimated at a timescale of ∼45,000 ya [25], although such estimation was mainly based on contemporary sampled sequences. Nevertheless, a recent survey of arenaviruses from non-mammalian hosts revealed large diversity of arenaviruses in reptiles (golden reptarenavirus and California reptarenavirus) [26,27] and ray-fined fish (Wenling frogfish arenavirus 1 and Wenling frogfish arenavirus 1) [27]. Evolutionary analyses including these viruses from divergent hosts revealed a striking similarity between the virus and host trees such that a co-divergence theory is strongly favoured for the entire family *Arenaviridae* [27]. In this study, the phylogenetic position of the newly identified arenavirus from Lagomorpha was sister to those identified from Rodentia, which was consistent with the evolutionary relationship of the hosts. Therefore, our findings further support the idea that arenaviruses co-diverge with their vertebrate hosts for billions of years, as the estimated divergence time of PPV was far earlier than that of existing mammarenaviruses.

Unlike the NW and OW mammarenaviruses, which infect rodents of the Sigmodontinae, Neotominae, and Murinae subfamilies, PPV can persistently infect plateau pikas of the Lagomorpha, which expands the host range of mammarenaviruses. Our results also provide direct evidence of extensive PPV host tropism in mammalian cell lines, including humans, primates, and rodents. PPV replication was similarly efficient in leporidae-originated RK-13 cells and human originated SLF-1 cells, a kind of embryo lung fibroblast diploid cell. Compared to RK-13 and SLF-1 cells, PPV showed reduced replication efficiency in Vero cells and A549 cells. The broad cell tropism may not indicate that PPV has a broad host range but will provide effective tools to further investigate the characteristics of PPV.

Previous studies have demonstrated that IFNαβR^-/-^ mice are susceptible to infection by mammarenaviruses (e.g. LASV and LCMV) and are commonly used in arenavirus pathogenicity [28–30]. We found that PPV could infect these mice, and the inflammatory infiltrates, edema in the cerebral cortex, and hemorrhage in the spinal cord upon intraventricular PPV challenge were observed, suggesting that PPV can apparently cause central nervous system-like lesions. However, similar nervous disease symptoms or lesions were not observed in animal models of SPF BALB/c mice, suckling mice and rabbits. Thus, our results indicate that PPV has potential pathogenicity in immunocompromised hosts.

Expanding human populations have a major impact on wildlife habitats, especially viral pathogens, accelerating the emergence of new infectious diseases through increased animal/human interactions [31]. Plateau pika is a keystone species of the Qinghai-Tibetan Plateau, with a high density of approximately 300 animals per hectare [32]. It has been reported that plateau pikas harbour zoonotic or related viruses, such as H5N1, H9N2, H7N2 and coronavirus [33–36]. Our retrospective serological survey showed that PPV antibodies were present in 2.4% of the serum samples from outpatients who lived in Yushu, Qinghai Province. We tried to detect viral RNA in the sera using specific primers against PPV and RNA-seq transcriptome analysis. No trace of PPV RNA was detected in any of the patient sera. However, this might be however inconclusive, as RNA degradation likely occurs in retrospective sera due to the long-term preservation. Serological surveillance in nearby inhabitants nearby is therefore needed. To strengthen the case for PPV as an agent of human disease, future surveys would ideally include a comparison of the seroprevalence in exposed and unexposed populations. LASV and LCMV cause more severe disease in immunocompromised individuals and pregnant women [9,37]. Therefore, these particular individuals should also be examined for PPV infection in the future.

In conclusion, we isolated a new mammarenavirus PPV from plateau pikaS in the Qinghai-Tibet Plateau. Our study elaborated on the characteristics, divergence history and potential infection of PPV. Its ability to replicate in IFNαβR^-/-^ mice and multiple cell lines revealed its potential infectivity. Antibodies against PPV present in human serum provide preliminary clues to the possibility of cross-species transmission to humans; thus, PPV may pose a risk to human health. Due to limited available samples, more wildlife and livestock samples (e.g. plateau pika, Himalayan marmot and yak) should be screened to assess how prevalent this virus is in the ecosystem. Furthermore, more microbiological and epidemiological studies are needed explore the prevalence of PPV in nature and evaluate its potential risk to human health.
