# Supplementary material for: Emergence of an ancient and pathogenic mammarenavirus
Source: Emerg Microbes Infect. 2023 Apr 11;12(1):e2192816. doi: 10.1080/22221751.2023.2192816 (PMC10337645; doi:10.1080/22221751.2023.2192816)
Supplement: Supplemental Material [file TEMI_A_2192816_SM3845.docx]

**Figure S1** **The virus load in different organs of five randomly selected plateau pikas with PPV infection.** Values are the averages of three independent experiments (mean ± SD).

**Figure S2 Maximum-likelihood phylogenetic trees of nineteen PPV genomes and known arenaviruses built on the basis of RdRp, GP and NP amino acid sequences.** Red indicates PPV sequences.

**Figure S3 Weight change in experimental animals infected with PPV.** (A) Weight change in New Zealand white rabbits infected with PPV via multipoint subcutaneous (sc) and intravenous (iv) inoculation. (B) Weight change in suckling mice infected with PPV via intraventricular (icv) and intraperitoneal (ip) inoculation. (C) Weight change in SPF BALB/c mice infected with PPV via intraventricular (icv) and intravenous (iv) inoculation.

**Figure S4 No pathogenic change was observed in the organs (heat, liver, kidney, lung and spleen) of the mice infected with PPV.** Those specimens were collected at 0 and 7 d.p.i. [scale bars: 0.5 mm.]


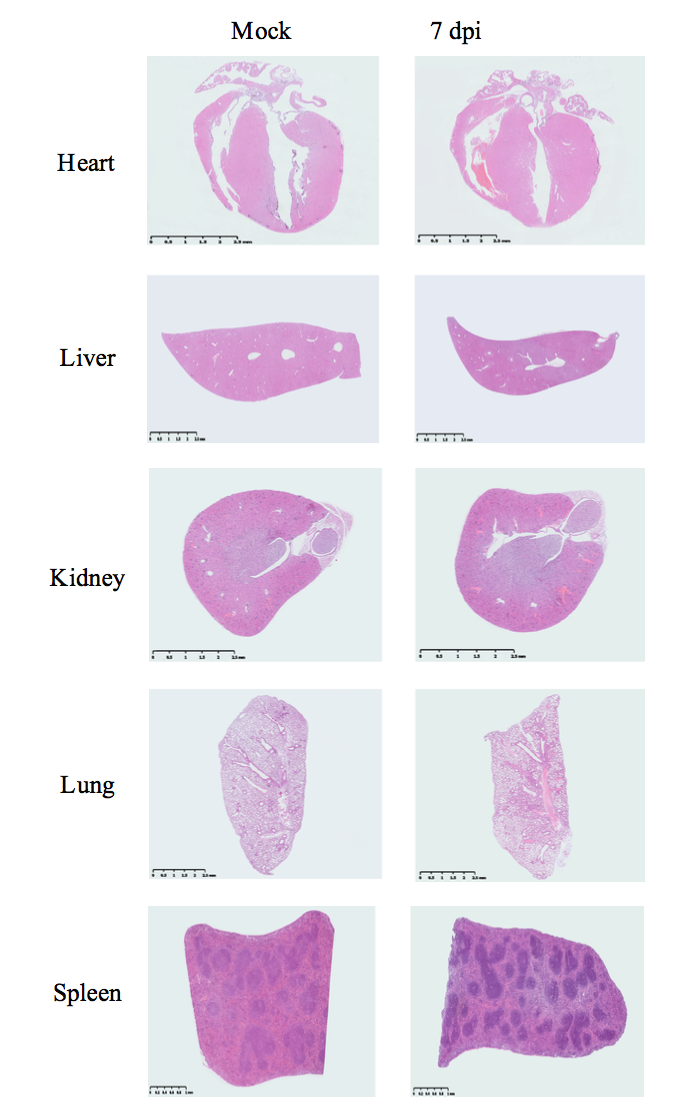


**Figure S5 Serum samples from the remaining seven patients who were IgG-positive against PPV confirmed by IFA and WB.** (A) Immunofluorescence images from seven patient serum samples, and a nonexposed individual. Human serum samples were diluted 1:40. (B) Western blot images from seven patient serum samples, and a nonexposed individual. Purified NPs (400 ng) of PPV, WENV and LCMV were used as antigens. Human serum samples were diluted 1:200.

**(A)**

**
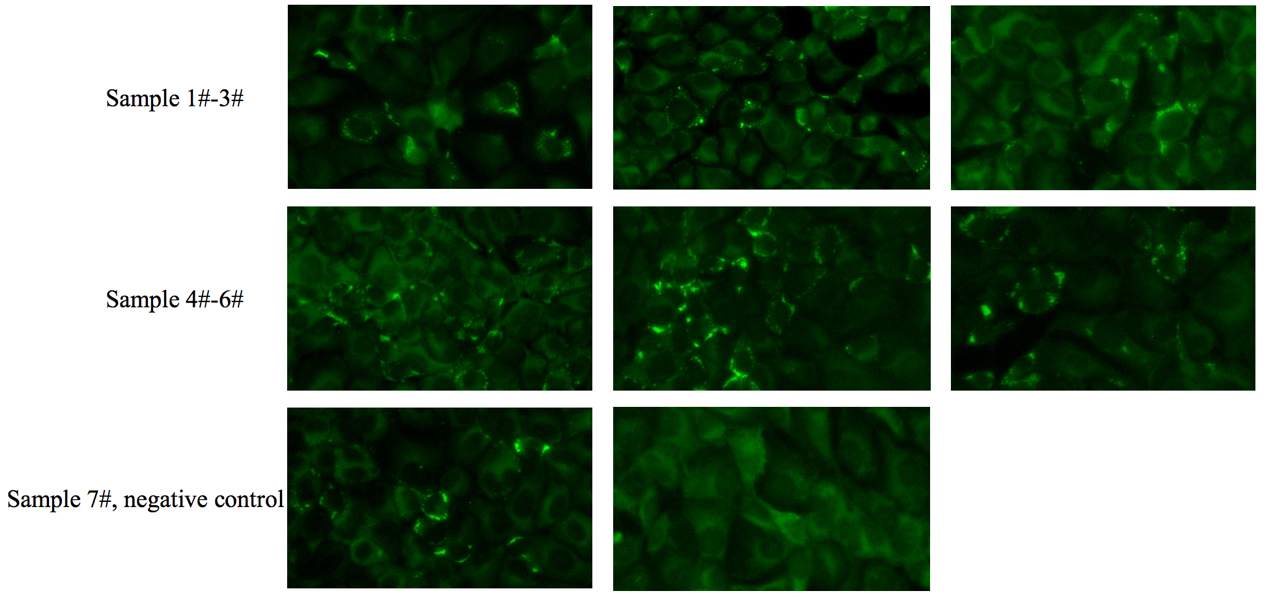
**

**(B)**

**
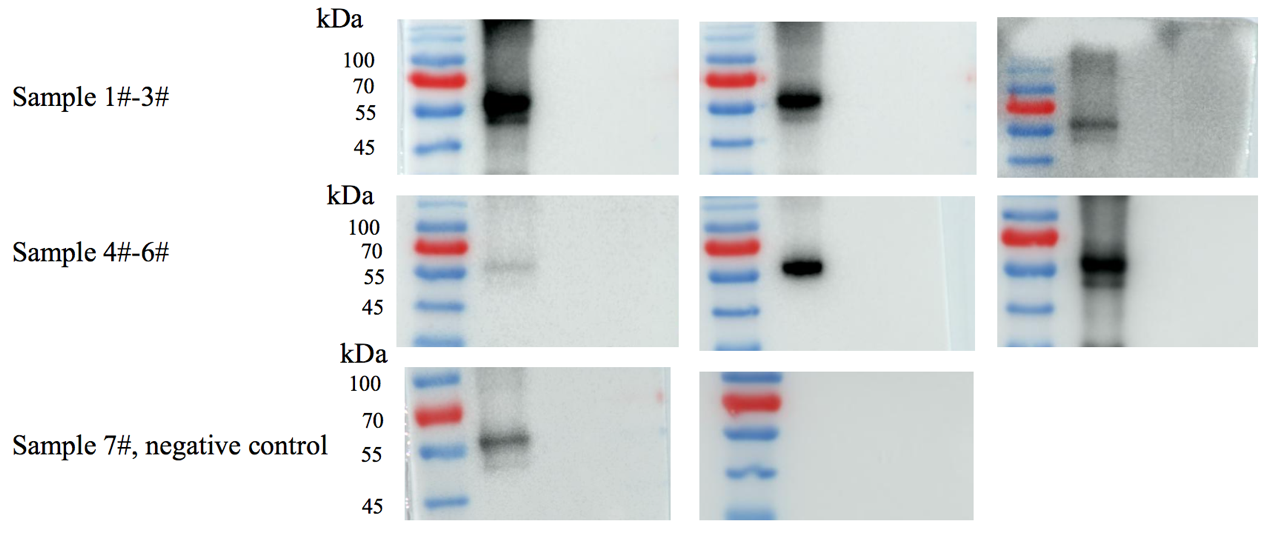
**

**Table S1 Table S1 List of primers used for confirmation of arenavirus contigs.**

|  | Sequence (5'->3') |  |
| --- | --- | --- |
| L-pairs 1(F12/R824) | CCCTCTGCTCCTGCACCGGG | for L segment |
|  | CACCTGTTCTATACTCTGATC |  |
| L-pairs 2(F854/R1858) | GTACTTCATCTTCAGTATATG |  |
|  | AGTTCTATATAGTCTCAAAAG |  |
| L-pairs 3(F1935/R2863) | GTGTGATGATGTTTTCTGAT |  |
|  | ATTTGGTCTGAGGAAGTGCC |  |
| L-pairs 4(F3358/4194) | GTGTTGACTTCATTCTTACTT |  |
|  | CACCTCGTTTACAAACACTAAG |  |
| L-pairs 5(F4666/R5495) | GAAGATGATCGAGGATCCTTAC |  |
|  | GGGCAACATTCCATACACTT |  |
| L-pairs 6(F6111/R6920) | CCCAACATACACTAAGTCTTT |  |
|  | TCAGAGCAATCCAGCCGCTG |  |
| S-pairs 1(F169/R904) | CATCCAAGCACTGATGTTCCT | for S segment |
|  | TCATAAGACAAAAGGTGAGAC |  |
| S-pairs 2(F1131/1939) | ACAAGCAGCTTGAAGTGGCAAC |  |
|  | CGAGGATGATGTGTGGAAGAA |  |
| S-pairs 3(F2217/R2887) | GCCTGTCGATGGTTGGAAGAC |  |
|  | TCAATTTGGGACAATGCCAGC |  |
| S-pairs 4(F2915/R3427) | GCAGGCTGGAATCTTTGATG |  |
|  | CATAGACTTAGTCTCTTTCGC |  |

# Table S2 Genomes of PPV found in *Ochotona curzoniae* and GenBank accession numbers.

| No. | Genome name | Length (nt) /Accession number | |
| --- | --- | --- | --- |
|  |  | L segment | S segment |
| 1 | O. curzoniae/YS8_2012/F | 7102/MN443982 | 3500/MN444002 |
| 2 | O. curzoniae/YS30_2012/F | 7084/MN443983 | 3542/MN444003 |
| 3 | O. curzoniae/YS46_2012/F | 7053/MN443984 | 3476/MN444004 |
| 4 | O. curzoniae/YS59_2012/F | 7106/MN443985 | 3498/MN444005 |
| 5 | O. curzoniae/YS45_2012/F | 7084/MN443986 | 3521/MN444006 |
| 6 | O. curzoniae/YS201_2015/F | 7108/MN443987 | 3515/MN444007 |
| 7 | O. curzoniae/YS1_2015/F | 7057/MN443988 | 3497/MN444008 |
| 8 | O. curzoniae/YS106_2015/F | 7081/MN443989 | 3487/MN444009 |
| 9 | O. curzoniae/YS224_2015/F | 7100/MN443990 | 3540/MN444010 |
| 10 | O. curzoniae/YS6_2012/F | 7107/MN443998 | 3482*/MN444018 |
| 11 | O. curzoniae/YS15_2012/F | 7411/MN443999 | 3529/MN444019 |
| 12 | O. curzoniae/YS27_2012/F | 7086/MN444000 | 3478*/MN444020 |
| 13 | O. curzoniae/YS68_2012/F | 7045*/MN443991 | 3461*/MN444011 |
| 14 | O. curzoniae/YS80_2012/F | 7043*/MN443992 | 3493/MN444012 |
| 15 | O. curzoniae/YS216_2015/F | 7084/MN443993 | 3544/MN444013 |
| 16 | O. curzoniae/YS400_2015/F | 7103/MN443994 | 3485/MN444014 |
| 17 | O. curzoniae/YS426_2015/F | 7084/MN443995 | 3511/MN444015 |
| 18 | O. curzoniae/YS490_2015/F | 7106/MN443996 | 3541/MN444016 |
| 19 | O. curzoniae/YS494_2015/F | 7094/MN443997 | 3501/MN444017 |

*indicates nearly complete sequences

**Table S3 Amino acid identities (%) of PPV with New World and Old World mammarenaviruses.**

|  | Virus | RdRp | NP | GP | ZP |
| --- | --- | --- | --- | --- | --- |
| New world | MACV Carvallo | 32 | 44 | 33 | 35 |
|  | JUNV XJ13 | 31 | 43 | 35 | 26 |
|  | TCRV | 31 | 43. | 32 | 33 |
|  | Chapare virus 810419 | 31 | 45 | 35 | 35 |
|  | SABV SPH114202 | 31 | 46 | 33 | 27 |
|  | CPXV BeAn 119303 | 31 | 46 | 38 | 29 |
|  | AMAV BeAn 70563 | 32 | 46 | 33 | 26 |
|  | GTOV INH-95551 | 32 | 46 | 35 | 27 |
|  | LATV MARU 10924 | 31 | 46 | 32 | 28 |
|  | OLVV 3229 | 31 | 42 | 32 | 36 |
|  | WWAV AV 9310135 | 33 | 45 | 35 | 35 |
|  | TAMV W 10777 | 33 | 42 | 34 | 39 |
|  | BCNV AV A0070039 | 33 | 46 | 37 | 35 |
|  | PARV 12056 | 33 | 47 | 33 | 31 |
|  | FLEV BeAn 293022 | 32 | 46 | 34 | 31 |
|  | PIRV | 32 | 47 | 34 | 35 |
|  | PICV AN3739 | 33 | 45 | 34 | 33 |
|  | ALLV VAV-488 | 33 | 46 | 34 | 31 |
| Old world | LUJV | 31 | 48 | 37 | 30 |
|  | Lunk virus NKS-1 | 31 | 44 | 37 | 31 |
|  | LCMV Armstrong 53b | 29 | 47 | 36 | 25 |
|  | MWV | 30 | 45 | 36 | 33 |
|  | Okahandja virus N73 OkhMi.n4 | 30 | 46 | 35 | 33 |
|  | IPPYV Dak An B 188d | 31 | 46 | 37 | 29 |
|  | Mariental virus N27 MRMi.n9 | 27 | 45 | 36 | 36 |
|  | Wenzhou virus Rn-242 | 31 | 46 | 36 | 35 |
|  | LASSV Josiah | 31 | 45 | 37 | 30 |
|  | MOBV Acar 3080 | 31 | 37 | 37 | 31 |
|  | Gairo virus TZ-27421 | 31 | 43 | 36 | 27 |
|  | Luna virus LSK-1 | 30 | 47 | 37 | 30 |
|  | MOPV AN20410 | 30 | 44 | 37 | 31 |
|  | Morogoro virus 3017/2004 | 30.8 | 44.2 | 36.8 | 31.2 |

**Table S4 Amino acid identity (%) among nineteen PPV sequences using MEGA7.0.** (A) L segment. (B) S segment.

**(A)**


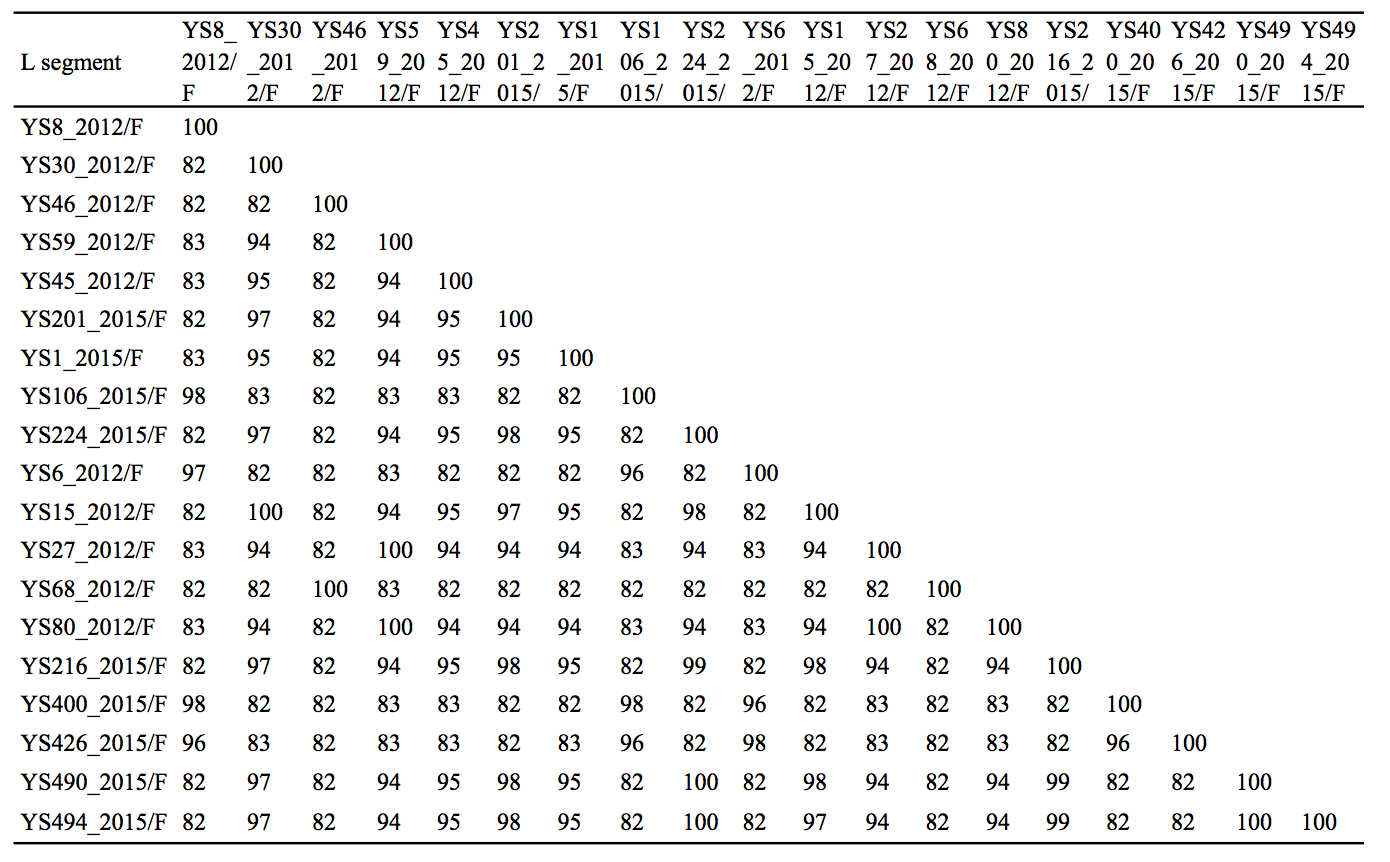


**(B)**


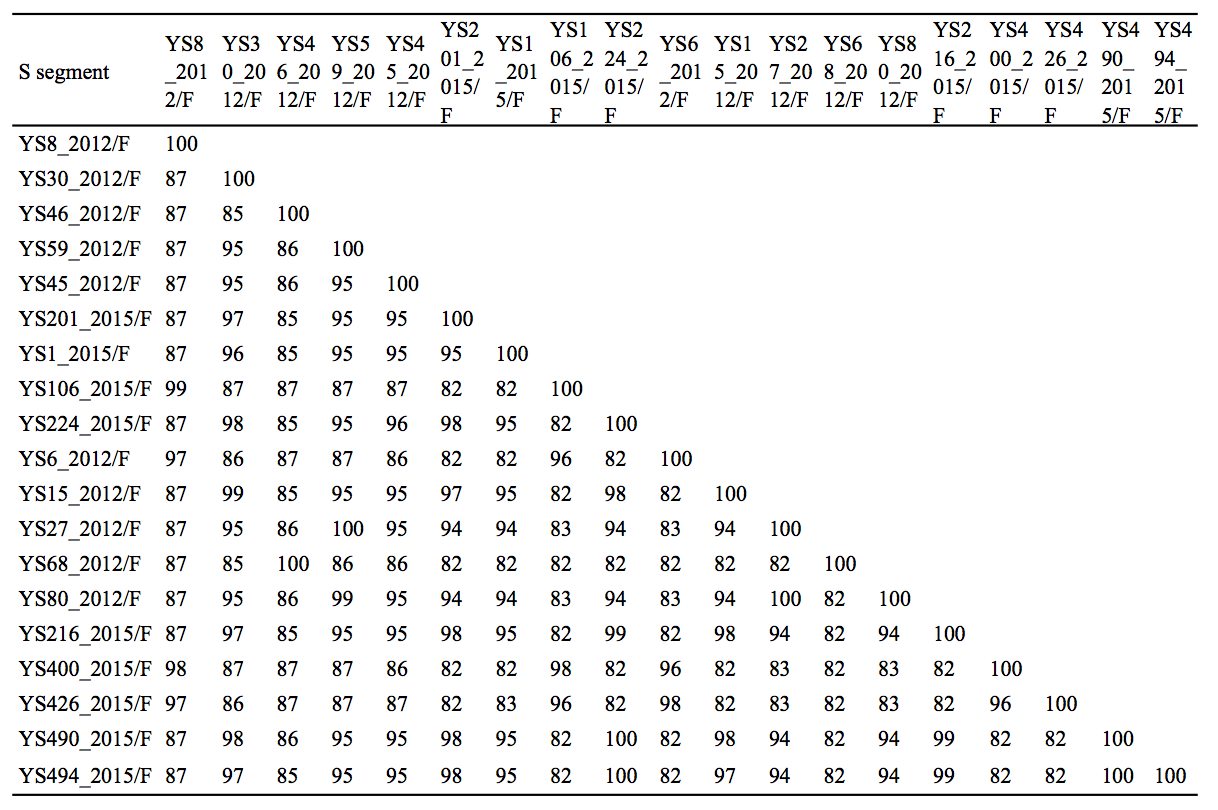


**Table S5 Detection of PPV RNA in organs of plateau pikas with PPV infection.**

|  | Lung | Kidney | Heart | Liver | Spleen | Brain |
| --- | --- | --- | --- | --- | --- | --- |
| Positive no. /No. of samples, percentage | | | | | | |
| *Ochotona curzoniae* | 34/34, 100 | 33/34, 97 | 25/26, 96 | 32/34, 94 | 24/25, 96 | — |
| 34/541, 6.3 |  |  |  |  |  |  |

— Not available

**Table S6 Sequence identity (%) between six PPV isolates using BioEdit 7.0.**

|  | Strain #8 | Strain #2 | Strain #11 | Strain #12 | Strain #4 | Strain #5 |
| --- | --- | --- | --- | --- | --- | --- |
|  | L segment /S segment | | | | | |
| Strain #8 | 100/100 |  |  |  |  |  |
| Strain #2 | 94/95 | 100/100 |  |  |  |  |
| Strain #11 | 85/82 | 85/82 | 100/100 |  |  |  |
| Strain #12 | 85/82 | 85/82 | 96/95 | 100/100 |  |  |
| Strain #4 | 95/95 | 97/98 | 86/82 | 85/82 | 100/100 |  |
| Strain #5 | 94/95 | 97/98 | 85/82 | 85/82 | 98/99 | 100/100 |
